# SLE Presenting With Unilateral Loculated Pleural Effusion in a Young Hypertensive Female: A Rare Case Report and Literature Review

**DOI:** 10.1002/ccr3.73497

**Published:** 2026-09-10

**Authors:** Amrit Tripathi, Ujwal Sah, Saurav Shah, Shivaditya Singh, Surya Raj Nishad

**Affiliations:** ^1^ Department of Medicine Manipal College of Medical Sciences Pokhara Nepal

**Keywords:** case report, hydroxychloroquine, lupus nephritis, SLE, unilateral loculated pleural effusion

## Abstract

SLE can rarely present with unilateral pleural effusion or hypertension. A 28‐year‐old woman with loculated exudative pleural effusion was diagnosed with SLE and lupus nephritis fulfilling EULAR/ACR criteria, treated with pigtail drainage, corticosteroids, and hydroxychloroquine, with good recovery. SLE should be considered in unexplained pleural effusion for timely diagnosis.

## Key Clinical Message

Unilateral loculated pleural effusion can be the initial manifestation of SLE in young patients. Early onset hypertension should prompt evaluation for lupus nephritis. Ultrasonography surpasses CT in detecting loculations. Timely multidisciplinary management with Hydroxychloroquine, corticosteroids, and surgical intervention ensures optimal outcomes.

## 1 Introduction

Systemic lupus erythematosus (SLE) is a chronic, autoimmune disease of unknown etiology with multiple systemic manifestations. Pleuropulmonary involvement is common, involving 20%–90% of cases. However, pleural effusion is a relatively uncommon presentation in the younger age group. The exact mechanism for pleuropulmonary involvement is not clear, though it is postulated to occur due to the presence of systemic interferons, circulating immune complexes, and neutrophils which trigger an inflammatory response in pleural and pulmonary epithelium resulting in a spectrum of disease [1]. There are eight types of pleuropulmonary involvement: lupus pleuritis, pleural effusion, acute lupus pneumonitis, shrinking lung syndrome, interstitial lung disease, diffuse alveolar hemorrhage, pulmonary arterial hypertension, and pulmonary embolism [2]. Clinical features vary to a wide range thereby creating a diagnostic challenge.

## 2 Case Presentation

### 2.1 Case History/Examination

A 28‐year‐old woman presented to the Emergency Department with a chief complaint of shortness of breath for 3 days which was acute in onset, aggravated on movement and also occurred at rest with no postural variation. The patient also complained of fever for 1 day, with a maximum recorded temperature of 103°F which was acute in onset, intermittent in nature and associated with chills and rigor.

On examination, the patient was anxious, ill‐looking with a fever of 101°F, regular pulse rate of 105 beats per minute, blood pressure of 160/90 mm of Hg and a peripheral arterial oxygen saturation of 98% on ambient air. Respiratory examination revealed reduced chest expansion on the right hemithorax on inspection and palpation. The trachea was centrally positioned, with no displacement of the cardiac apex. Tactile vocal fremitus was decreased over the right lower lung fields. Percussion elicited a dull note over the right infrascapular, infra‐axillary, and lower mammary regions. Auscultation demonstrated markedly diminished breath sounds, with decreased vocal resonance over the affected area, consistent with a right‐sided pleural effusion.

### 2.2 Investigations and Treatment

**FIGURE 1:**
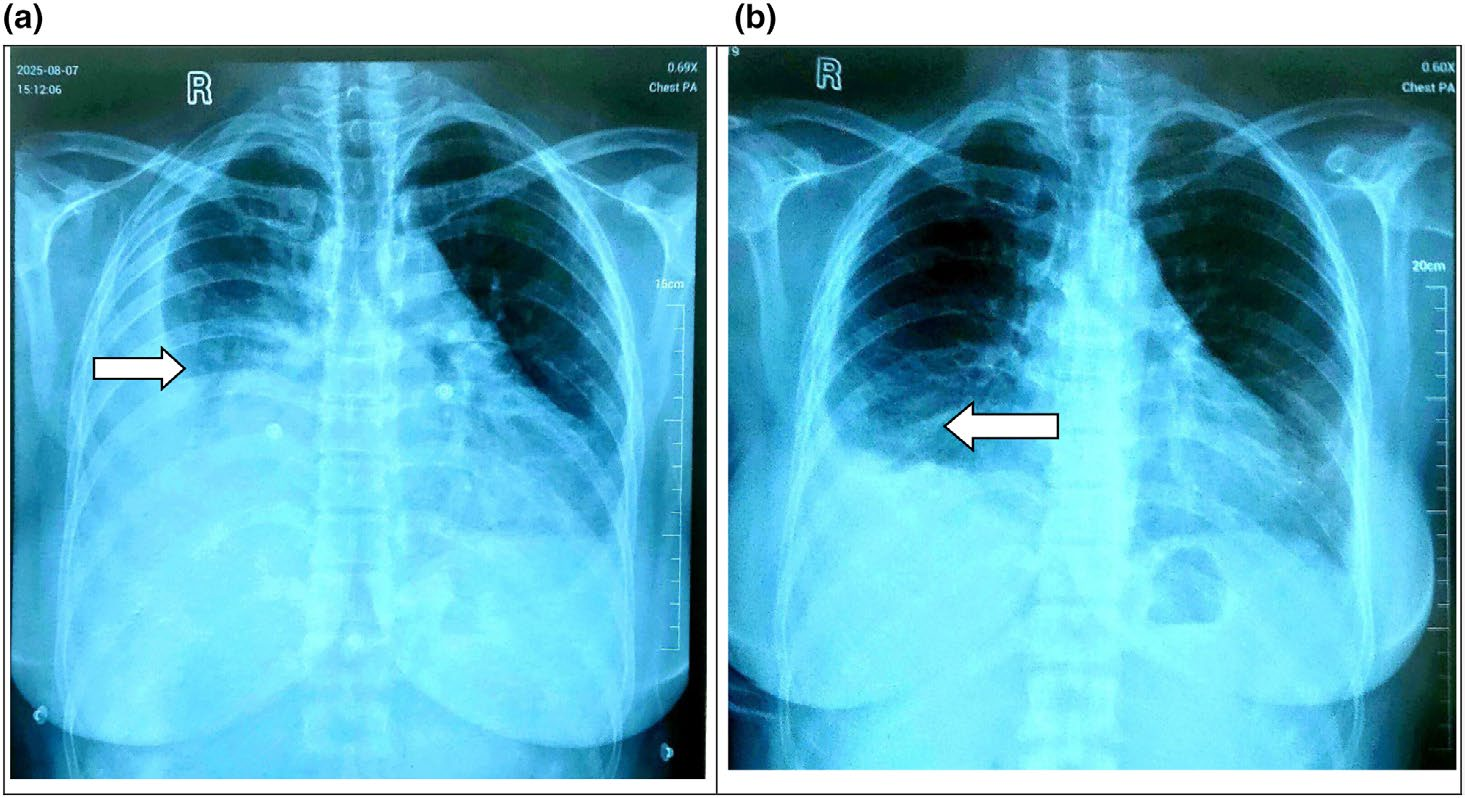
Chest X‐ray revealed blunting of right costophrenic angle with meniscus shaped fluid filled level during admission (a) and at the time of discharge (b).

Laboratory tests revealed microcytic hypochromic anemia (hemoglobin‐ 8.7 g/dL, MCV‐ 66 fl) and elevated C‐reactive protein. Iron profile revealed normal serum ferritin (24.7 ng/mL) with normal total iron binding capacity (259 mcg/dL). Chest X‐ray revealed blunting of right costophrenic angle with meniscus‐shaped fluid‐filled level (Figure 1a).

**FIGURE 2:**
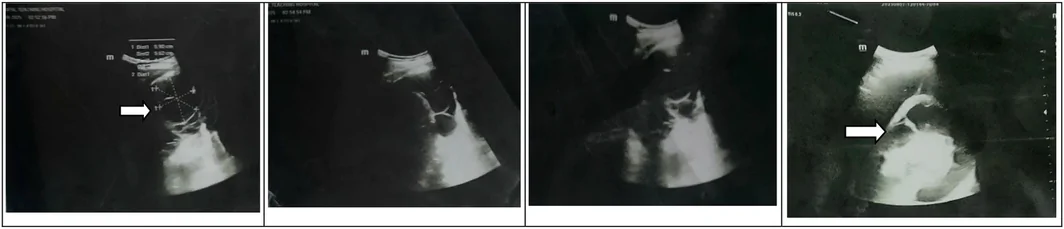
USG chest showed heterogeneously echogenic fluid with punctate hyperechoic foci in right pleural space suggestive of complex septated right pleural effusion.

**FIGURE 3:**
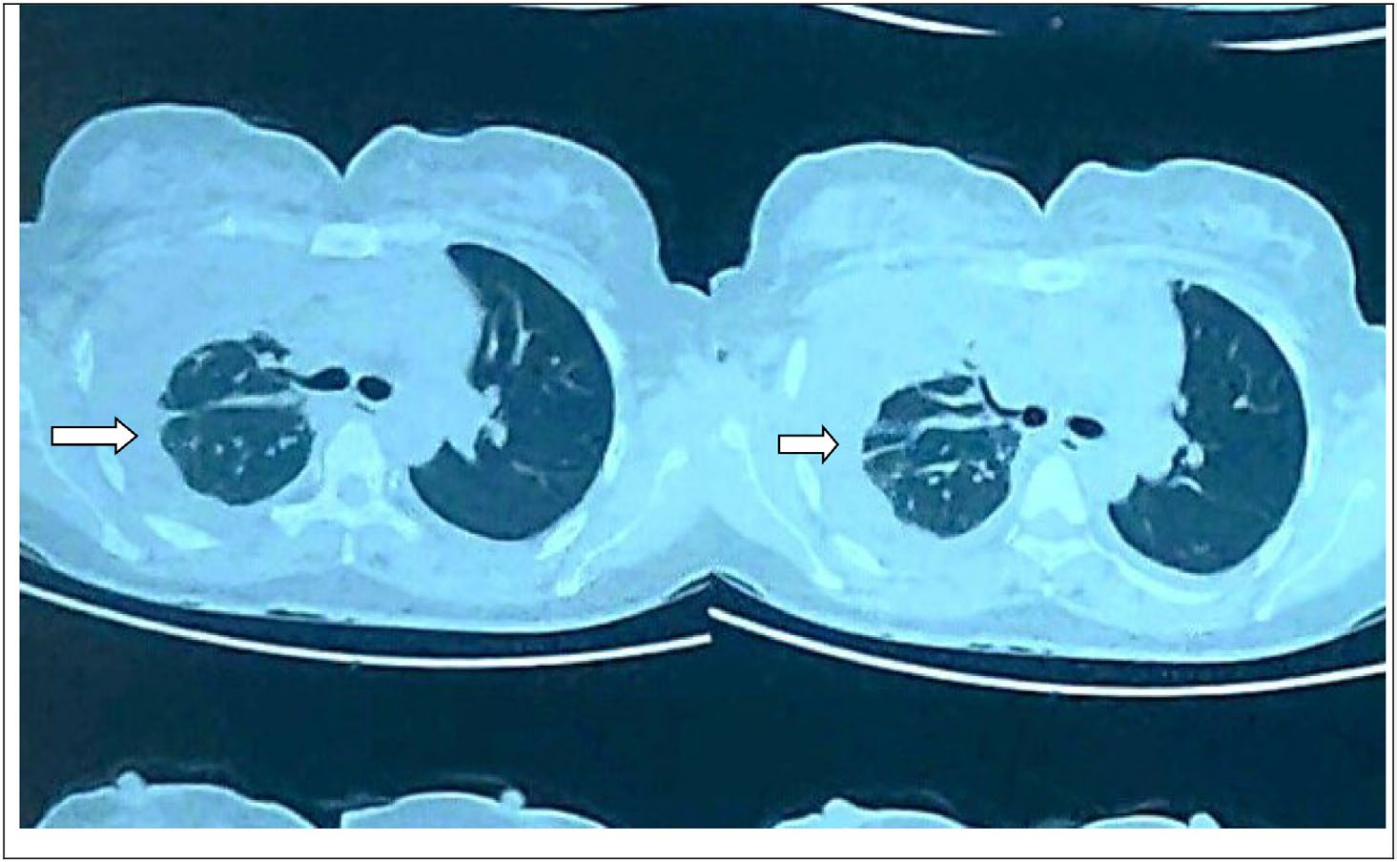
CECT scan of chest revealed gross right pleural effusion with passive collapse of underlying lung parenchyma and lower mediastinal shift to left side.

Renal Function tests, liver function tests, thyroid function tests, and arterial blood gas analysis were within normal limits. Echocardiography showed normal cardiac anatomy with normal ejection fraction. Serology for HIV, Hepatitis B and C virus, Leptospira, Brucella, Dengue, Malaria was nonreactive. Sputum Gram stain and Ziehl–Neelsen (AFB) stain were negative, and blood and sputum cultures showed no growth. Ultrasonography (USG) of the chest showed heterogeneously echogenic fluid with punctate hyperechoic foci in the right pleural space suggestive of complex septate right loculated pleural effusion (Figure 2). Contrast Enhanced Computed Tomography (CECT) scan of the chest revealed gross right pleural effusion with passive collapse of underlying lung parenchyma and lower mediastinum shift to the left side (Figure 3).

Pigtail catheter was inserted in right pleural space; pleural fluid was aspirated and sent for investigations (Table 1) and streptokinase fibrinolysis was done for loculated pleural effusion. Pleural fluid analysis revealed low glucose, elevated total protein, and elevated LDH, with a normal ADA level and normal cytological findings on smear preparation. Pleural fluid Gram stain and Ziehl‐Neelsen stain were negative, and pleural fluid as well as sputum cultures showed no bacterial growth after 48 h of incubation. Application of Light's criteria confirmed an exudative process: the pleural fluid LDH was more than two‐thirds of the upper limit of normal serum LDH, and the pleural fluid‐to‐serum protein ratio was elevated (> 0.6).

**TABLE 1:** Baseline investigations and pleural fluid analysis.

Parameters	Values	Reference
Total WBC	9400	4000–11,000
Neutrophils	70	40–80
Lymphocytes	22	20–40
Hemoglobin	8.7	13–17
Platelet count	136,000	150,000–400,000
MCV/MCH/MCHC	66/23/33	83–100/27–32/31–35
Serum creatinine/urea	0.52/11	0.3–1.3/10–45
Serum sodium/potassium	137/3.98	135–145/3.5–5.1
Total protein/albumin	6.0/2.7	6.0–8.2/3.5–5.0
Total bilirubin	0.7	0.2–1.3
AST/ALT/ALP	16/34/71	5–35/5–45/38–126
Serum ferritin/TIBC/serum iron	24.7/259/17	6.24–137/250–425/50–170
Reticulocyte count	0.4	0.2–2
CRP/ESR	92.0/50	0–10/0–20
Free T3/Free T4/TSH	2.87/1.65/2.09	2.77–5.27/0.78–2.19/0.46–4.68
pH	7.45	7.60–7.64
Glucose	51	60–100
LDH	906	93–187
ADA	35	0–40
Total protein	4.9	< 1.5
Pleural fluid protein:serum protein	0.72	Indicated exudative pleural effusion (as per Light's criteria)
Pleural fluid LDH:serum LDH	4.84 (> 2/3 of upper limit of normal serum LDH)	Exudative pleural effusion
Pleural fluid cytology	80% lymphocytes and 20% neutrophils with few reactive mesothelial cells in small clusters; no atypical cells	
Gram stain	No organisms detected in blood/sputum/pleural fluid	
ZN stain	No organism seen in sputum/pleural fluid	
Culture and sensitivity	No growth after 48 h of incubation in blood/sputum/pleural fluid	

Several differential diagnoses were considered given the loculated nature of the effusion and associated mediastinal shift. Empyema was considered unlikely in the absence of purulent fluid on thoracocentesis or organism growth on Gram stain and culture. Tuberculosis was considered less likely based on the clinical, biochemical, microbiological, and radiological findings. Malignancy was considered less likely, given the absence of pleural nodularity, pleural thickening, mass lesion, or mediastinal or hilar lymphadenopathy on CECT chest, and pleural fluid cytology showing no atypical or malignant cells. The Wells score was 1.5, corresponding to low clinical probability, with tachycardia (105 beats per minute) as the only contributing variable. Pulmonary embolism was therefore considered clinically unlikely. D‐dimer estimation and CT pulmonary angiography were not performed, as the dyspnea was attributable to a radiologically confirmed large loculated effusion with passive collapse and mediastinal shift, and resolved following pleural drainage. Cardiac causes of effusion were considered unlikely given the unilateral, loculated nature of the fluid, which is atypical for a transudative process such as heart failure, and this was further supported by a normal echocardiogram and Light's criteria confirming an exudative effusion.

On detailed retrospective workup of the patient, it was found that she had a history of hypertension for 9 years, and recurrent photosensitive rashes over sun‐exposed areas, arthralgia, non‐scarring hair loss, and recurrent aphthous oral mucosal ulcers for approximately 2 years prior to admission; the cause of which was not evaluated in the past. Therefore, ANA titre and ENA panel were sent, and results are shown in Table 2. Serum C3, C4 and urine investigations were sent and results are depicted in Table 3. A clinical diagnosis of SLE was made on the basis of these features together with the serological findings and was supported by the 2019 EULAR/ACR classification criteria. The entry criterion of a positive ANA (> 1:80 on HEp‐2 cells) was met, with additional domains scoring as follows: fever (2), non‐scarring alopecia and oral ulcers (2), serositis in the form of pleural effusion (5), renal involvement with proteinuria of 900 mg/day (4), low C3 and C4 (6), and anti‐Sm antibody (6), giving a cumulative score of 25 against a classification threshold of 10 points. Taken together, the findings indicated an exudative effusion secondary to lupus serositis rather than an infectious, tuberculous, or malignant process.

**TABLE 2:** ENA panel.

ANA 1:640 titre (nuclear large/coarse speckled)	
Antibody against	Intensity
Anti‐ds DNA	3+
Nucleosome	2+
Histone	1+
RNP/Sm	3+
Sm	3+
Mi‐2α	1+
Mi‐2β	3+
K4	1+
Centromere	3+
PM‐Scl100	2+
PM‐Scl75	3+
PCNA	1+

Qualitative result	Explanation
1+	Borderline positive
2+	Positive
3+	Moderate positive

**TABLE 3:** Renal workup for SLE.

Parameter	Result	Normal range
Serum C3	65 mg/dL	90–180 mg/dL
Serum C4	7 mg/dL	20–50 mg/dL
CK‐MB	0.01	0–16
Urine protein	29.6	< 12
24 h urine protein	900 mg/day	42–225

**FIGURE 4:**
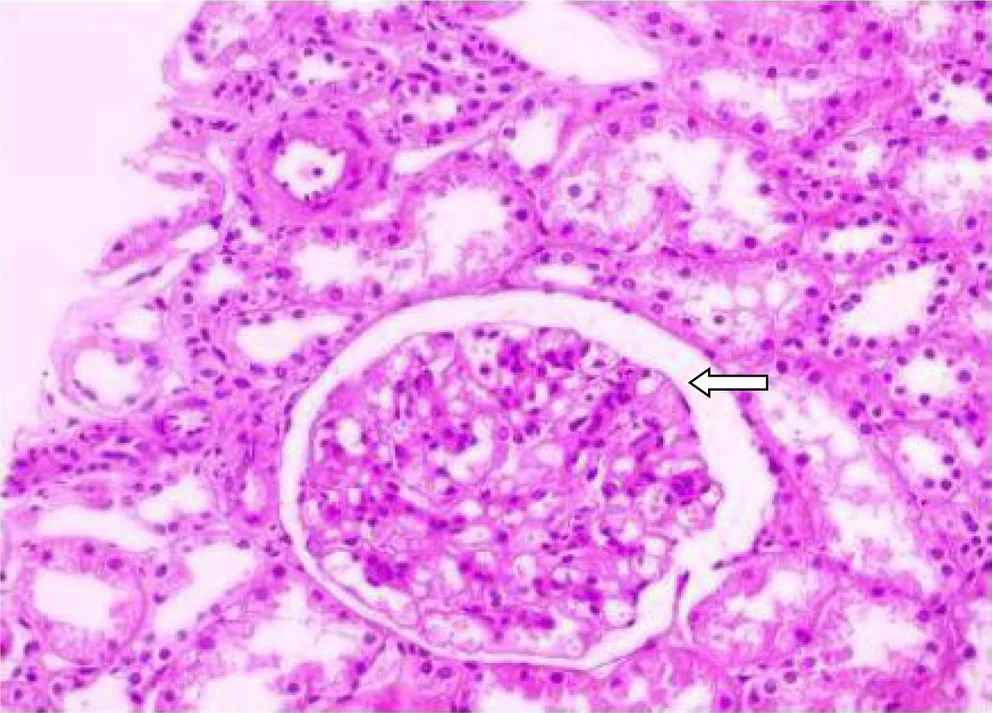
Renal biopsy revealed multiple sections stained with H&E stain, which showed small renal cortical parenchymal core. The glomeruli appear enlarged and reveal a diffuse mild increase in mesangial matrix and cellularity, and diffuse thickening of capillaries, which show membrane texture alterations with intramembranous “mottling” and few epimembranous “spikes.” One glomerulus shows segmental endocapillary hypercellularity. Segmental tuft sclerosis, crescent formation, or active tuft necrosis are not observed. Tubular atrophy and interstitial fibrosis involve < 10% of the sampled cortex. Tubules show focally prominent cytoplasmic vacuolar change. Scattered hyaline casts are seen in tubular lumina, and focal chronic interstitial inflammation is noted.

Following the diagnosis of lupus pleuritis, a renal biopsy was planned to further evaluate renal involvement in the patient. Renal biopsy showed glomeruli, which appeared enlarged and revealed diffuse increase in mesangial matrix mass and cellularity with diffuse thickening of capillaries which showed membrane texture alterations with intermembranous mottling and few epimembranous spikes. Tubular atrophy and interstitial fibrosis involved < 10% of sampled cortex with focally prominent cytoplasmic vacuolar change and scattered hyaline cast in tubular lumen. Tissues for direct immunofluorescence showed granular immunostaining pattern in capillary wall and mesangium. The overall picture was indicative of coexisting lesions of Membranous lupus nephritis: ISN/RPS (2018) Class V and Focal lupus nephritis: ISN/RPS (2018) Class III (focal lupus nephritis); with indices (NIH) of activity 2/24 and chronicity 0/12 (Figure 4). The patient's young‐onset hypertension was evaluated for secondary causes. Renal ultrasound and renal doppler sonography did not reveal any structural abnormality suggestive of renovascular cause, and thyroid function tests were within normal limits. There was no history of oral contraceptive use, NSAID use, or other medications. Family history was noncontributory for hypertension or renal disease. Given the presence of proteinuria and renal biopsy findings, lupus nephritis was considered a possible contributor to her hypertension, although the etiology could not be established retrospectively.

Upon arrival to the hospital, after primary treatment at Emergency Room (ER), the patient was transferred to the ICU, where a pigtail catheter was inserted into the right pleural space and intrapleural fibrinolysis was performed with streptokinase 3,00,000 IU diluted in 50 mL of normal saline, instilled over 20 min. The procedure was uneventful and well tolerated, with no complications, and drainage improved subsequently with radiological resolution as shown in Figure 1b. The patient was treated with empirical intravenous antibiotics, antihypertensive drugs, IV proton pump inhibitor (PPI) and diuretics. After discussion with nephrology, the patient was started on oral prednisolone 40 mg, which was gradually tapered following clinical remission in 3 weeks and daily oral hydroxychloroquine alone. The renal disease appeared histologically quiescent with focal proliferation, NIH activity index 2/24, chronicity 0/12, interstitial fibrosis under 10%, with sub‐nephrotic proteinuria of 900 mg/day, normal creatinine (0.52 mg/dL), and no features of rapidly progressive glomerulonephritis. Her dominant problem was serositis rather than nephritis. Cost, monitoring requirements, and the broad adverse effects in a woman of reproductive age also weighed on the decision, and thus no further immunosuppressive agent was started at that stage.

### 2.3 Outcome and Follow‐Up

Patient was discharged after 3 weeks of hospital stay, following significant resolution of right loculated pleural effusion as evidenced by Chest X‐ray, shown in Figure 1b. She has remained under regular clinical follow‐up for 2 years, most recently reviewed within the past 3 months. She has experienced intermittent episodes of pleuritis, each of which resolved with supportive treatment during brief hospital stays. Frequent relapses were managed with short courses of oral corticosteroids, alongside continued maintenance therapy with hydroxychloroquine. The patient remains asymptomatic and continues her daily activities without limitation, between episodes. Follow‐up was clinical and investigations‐based; serum creatinine, proteinuria, complement levels, anti‐dsDNA titres, and repeat chest imaging were within normal limits. The entire timeline of progression from presentation to diagnosis and treatment of the patient is depicted in Table 4.

**TABLE 4:** Timeline table of progression from presentation to diagnosis and treatment of the patient.

Timepoint (in day)	Events
Day 0	Young hypertensive female presents with acute‐onset symptoms; fever and shortness of breath. Clinical examination reveals unilateral reduced breath sounds; hypertension noted; initial infection‐like presentation suspected
Day 1	CXR and USG chest reveal unilateral loculated pleural effusion, CECT showed unilateral right pleural effusion with mediastinal shift
Day 2	Thoracentesis performed; pleural fluid analysis shows exudative effusion (low glucose, elevated protein/LDH, normal ADA)
Day 3–5	Gram stain and pleural/sputum cultures sent (negative at 48 h); acid‐fast stain performed on sputum and pleural fluid‐ showed negative results. Infectious and malignant causes ruled out based on culture, ADA, and imaging findings
Day 6	Based on past history suggestive of SLE‐ rash, alopecia, arthralgia; Serological workup sent‐ANA (HEp‐2), anti‐dsDNA, anti‐Sm, C3/C4, urinalysis for proteinuria
Day 7–10	ANA titre > 1:80; low C3/C4; anti‐Sm positive; proteinuria 900 mg/day; SLE diagnosis confirmed per 2019 EULAR/ACR criteria (score: 25 points)
Day 10–14	Treatment initiated with corticosteroids for acute presentation, hydroxychloroquine started on maintenance therapy; Clinical improvement; pleuritis symptoms resolve; discharged
Follow‐up	Regular follow‐up; intermittent pleuritis relapses managed with short corticosteroid courses; hydroxychloroquine continued; asymptomatic between episodes

## 3 Discussion

Systemic Lupus Erythematosus (SLE) is a multisystem disease of unknown etiology. It involves formation of autoantibodies against various nuclear antigens [3]. These antibodies and their immune complexes are responsible for various clinical manifestations of SLE which predominantly affect young females. Studies have suggested that incidence of pleural effusion in SLE increases with age, with higher prevalence among older patients as compared to younger patients. Advancement in age alters the expression of SLE in terms of clinical presentation (pleuritis and pericarditis being the most common presenting complaint) and pattern of organ involvement (pulmonary involvement being more common as compared to lymphadenopathy, neuropsychiatric disorder, alopecia, or skin disease) [4] In contrast to these findings, our patient developed pleural effusion due to SLE at a considerably younger age. Around 4%–5% of patients present with initial pulmonary manifestation. There is a wide range of bronchopulmonary spectrum ranging from mild lupus pleuritis to very severe shrinking lung syndrome, with lupus pleuritis being the most common manifestation [5]. Pleural Effusion in SLE can occur due to primary causes such as lupus pleuritis, or due to secondary causes like infection, heart failure, pulmonary embolism, or renal failure [6].

SLE is well known for its diverse and often unpredictable clinical spectrum, and rare, atypical presentations have been documented in the literature. These include visual disturbances such as homonymous hemianopia [7], acute confusional states as a neuropsychiatric manifestation [8], gastrointestinal involvement in the form of esophageal motor dysfunction and aperistalsis [9], and massive pericardial effusion as an initial presenting feature [10]. Such reports underscore the importance of maintaining a high index of suspicion for SLE across a wide range of atypical presentations, particularly when initial features mimic more common infectious or systemic conditions, as seen in the present case.

Patients usually present with shortness of breath, pleuritic chest pain, fever, and cough as seen in our case. Examination findings showed features of right‐sided pleural effusion. Pleural effusion secondary to SLE is usually bilateral and usually mild to moderate in size, but our patient presented with unilateral moderate‐to‐severe pleural effusion, and more rarely with loculated pleural effusion [11]. Chest CT scan revealed gross right pleural effusion with collapse of underlying lung parenchyma and shifting of lower mediastinum to left side as well, with no loculations. However, USG of chest showed presence of heterogeneous echogenic fluid with multiple punctate foci in right pleural space suggestive of complex septate loculated pleural effusion. This discordance reflects a recognized limitation of CT rather than a contradiction: fibrinous septa are thin and of attenuation close to that of the surrounding fluid, so they frequently fall below the contrast resolution of CT, whereas ultrasound resolves them readily in real time. In a recent comparative study using medical thoracoscopy as the reference standard, ultrasound identified septated effusions with a sensitivity of 82.6% compared with 59.8% for contrast‐enhanced CT [12]. In our patient, loculation was further supported functionally by incomplete drainage through the pigtail catheter, which improved following intrapleural streptokinase.

Pleural fluid in SLE is an exudative type which occurs due to increased permeability of the mesothelial membrane resulting in the entry of protein and cells into the pleural space or due to impaired removal of cells and protein. Exudative pleural effusion is defined according to Light's criteria [13]. Loculated pleural effusion can result from intense chronic pleural inflammation which recruits lymphocytes and fibroblasts to the site of inflammation resulting in the formation of adhesions and bands thus trapping pleural fluid. Various causes of loculated pleural effusion include empyema, hemothorax, tuberculosis, or malignancy [14] Microbiological investigations and pleural fluid cytology argued against an infectious cause for the loculations in our case. Unilateral pleural effusion due to SLE is extremely rare, with few case reports existing in the literature. To the best of our knowledge, loculated unilateral pleural effusion as a presenting feature of SLE is a rare presentation, and we identified no previously reported cases in our search of the published literature.

Diagnostic pleural tapping is essential to intervene for the cause of unilateral pleural effusion. As per the prospective study conducted by Syed Ali Abbas et al. [15], 48 patients with unilateral effusion were identified; out of which 54% had tuberculosis, 20% malignancy, 8% left ventricular failure, 6% chronic liver disease, 4% parapneumonic effusion, and 7% with unknown cause. Less common causes include kidney disease, asbestos exposure, lupus pleuritis, pancreatitis, and cardiac surgery [14]. Biochemical analysis revealed high protein (pleural fluid protein/serum protein—> 0.5), high LDH, and normal to low glucose level which is suggestive of exudative fluid as per Light's criteria. Cell cytology of pleural fluid showed lymphocytes (80%) and neutrophils (20%) with few reactive mesothelial cells in small clusters. Pleural fluid ADA was < 40 U/L; Ziehl‐Neelsen and Gram stains were negative on both pleural fluid and sputum; cultures of pleural fluid, sputum, and blood showed no growth, and serology for other causative organisms was nonreactive. Similarly, echocardiography with findings of normal cardiac function and normal ejection fraction helped in ruling out overt cardiac dysfunction. Taken together, these findings argued strongly against a tuberculous, other infectious, or cardiac etiology, although they did not permit definitive exclusion of tuberculosis [16].

On further exploration, patient had history of recurrent photo‐sensitivity rashes, arthralgia, alopecia and hypertension for 9 years, which raised suspicion of autoimmune causes like SLE. ANA titre of 1:640 on the background of underlying history supported diagnosis of SLE as a cause for unilateral pleural effusion as per 2019 EULAR/ACR criteria. EULAR/ACR criteria requires an entry test of positive ANA titre (> 1:80 on HEp‐2 cells), satisfied with additional clinical (constitutional, cutaneous, arthritis, neurological, serositis, hematological and renal domains) and immunological (antiphospholipid antibody, complement protein a and highly specific antibody domains). Each domain is assigned a weighted score, and a cumulative total of ≥ 10 points in the presence of a positive ANA permits classification as SLE [17]. These criteria were developed and validated for classification in research settings, with reported sensitivity and specificity of approximately 96% and 93% respectively, which supports clinical diagnosis [17].

As per Palavutitotai et al. [18], out of 119 patients diagnosed with pleural effusion in SLE, causes include lupus pleuritis (52%), tuberculous pleuritis (9%), parapneumonic effusion (7%), and transudate (15%). The diagnosis of lupus pleuritis was based on clinical diagnosis (47%) and by excluding other causes based on pleural fluid analysis or biopsy (53%).

A cautious interpretation of our microbiological findings is warranted in a high‐burden tuberculosis setting. Acid‐fast microscopy is positive in under 5% of tuberculous pleural effusions and mycobacterial culture in only about one‐third [19], while ADA, the most sensitive noninvasive marker (pooled sensitivity 92%, specificity 90% at 40 U/L) can be negative in culture‐confirmed disease [20]. Our patient's value of 35 U/L lay close to the cutoff, and the lymphocyte‐predominant, low‐glucose exudate observed here is itself compatible with tuberculous pleuritis. Pleural biopsy and nucleic acid amplification testing, the more sensitive confirmatory modalities, were not performed, as the serological and renal findings established a diagnosis of SLE that accounted for the clinical picture and the patient improved rapidly on immunosuppression.

On diagnosing lupus pleuritis as a cause for unilateral loculated pleural effusion, nephrology consultation was done. Extractable nuclear antigen panel showed elevated antibody levels against antigens (dsDNA, Sm, Centromere). Serum C3 and Serum C4 counts were low, indicating active lupus flare [21]. Urine protein‐to‐creatinine ratio and 24 h Urine protein were raised, which is suspicious of lupus nephritis. Following these findings, renal biopsy was done, which showed enlarged glomeruli with an increase in mesangial matrix and cellularity as well as diffuse thickening of the membrane indicative of coexisting lesions of membranous and focal lupus nephritis.

The patient had a 9‐year history of hypertension that had not previously been evaluated. Hypertension is common in lupus, with a reported prevalence of up to 74%, and Mbengue et al. found hypertension in 23 of 64 patients (43.75%) with lupus nephritis [22]. Mechanistically, proliferation of mesangial matrix and glomeruli in lupus can result in decreased renal perfusion, which activates Renin‐Angiotensin‐Aldosterone System (RAAS), thereby increasing release of angiotensin II which results in Hypertension [23]. In our patient, however, the temporal relationship remains uncertain: the hypertension preceded her first recognized lupus manifestations by several years; no prior renal or blood pressure workup was available for comparison, and the biopsy showed no chronic damage (chronicity index 0/12, interstitial fibrosis < 10%), which would be unexpected had lupus nephritis been present throughout that period. Secondary causes were nonetheless addressed at presentation, with renal ultrasound and Doppler showing no renovascular or structural abnormality, normal thyroid function, and no contributory drug or family history. We therefore consider the etiology of her hypertension undetermined, with lupus nephritis a plausible contributor rather than an established cause, and would emphasize that early‐onset hypertension in a young woman warrants evaluation for secondary causes irrespective of the eventual diagnosis.

Our patient also had microcytic hypochromic anemia (hemoglobin 8.7 g/dL, MCV 66 fL) with an elevated CRP of 92 mg/L. Serum iron was low at 17 μg/dL with a TIBC of 259 μg/dL, giving a transferrin saturation of approximately 7%, and serum ferritin was 24.7 ng/mL without any significant bleeding history. The reticulocyte count of 0.4% was inappropriately low for the degree of anemia. Ferritin is an acute‐phase reactant and is typically elevated during active inflammation; a value in this range alongside a markedly reduced transferrin saturation therefore indicates true iron depletion rather than the functional iron restriction of anemia of chronic disease alone. The low‐normal TIBC, which would be expected to rise in uncomplicated iron deficiency, reflects the concurrent inflammatory state. Taken together, the findings are consistent with iron deficiency anemia superimposed on anemia of chronic disease in the setting of active SLE.

Treatment of pleural effusion was started with conservative management which included IV antibiotics, IV PPI, and oral antihypertensive drugs. Upon arriving at the diagnosis of SLE, the patient was started on steroid therapy and oral hydroxychloroquine. Treatment options include NSAIDs, glucocorticoids, and immunosuppressive agents. Currently, Hydroxychloroquine has been the background drug for SLE if there are no contraindications [24]. It reduces renal flare‐ups, improves endothelial function, and prevents platelet aggregation thereby reducing cardiovascular risk [4]. It has proven benefit against bacterial and viral infections. Risk of maculopathy has been reported on long‐term use. A high dose of steroids, mainly methylprednisolone, can alleviate the symptoms of flare [25]. Other immunosuppressive agents include mycophenolate, azathioprine, and cyclosporine. Biological agents like Rituximab and more recently, Belimumab have demonstrated benefit. For patients with an inadequate response to standard therapy, Belimumab has shown good outcomes especially as an add‐on therapy in patients with active, antibody‐positive SLE [26]. For resistant cases, treatment with intravenous immunoglobulin can be considered [27].

Surgical intervention includes pigtail catheter insertion and streptokinase injection, which helps in adhesiolysis of loculated pleural effusion, which is consistent with our case [28]. Other surgical treatment modalities include talc pleurodesis and pleurectomy. Talc pleurodesis is preferred when systemic measures fail or pleural manifestation is the sole manifestation of lupus [29].

Compared with previously reported cases, our patient differs in several respects. Tsuji et al. described unilateral effusion as the initial manifestation of SLE, but in a patient of advanced age and without loculation [3], whereas our patient presented at 28 years, an age at which pleural involvement is less commonly the presenting feature. Khatieb et al. reported massive unilateral effusion requiring rituximab for control [11], whereas ours responded to corticosteroids and hydroxychloroquine combined with catheter drainage and intrapleural fibrinolysis, without escalation to biologic therapy. In the series by Palavutitotai et al., lupus pleuritis accounted for just over half of effusions in SLE, though loculation was not a described feature [18]. Renal involvement is likewise not consistently reported in these cases, whereas our patient had biopsy‐proven mixed Class III and V lupus nephritis identified only after the pleural presentation. The combination of unilateral loculation, biopsy‐confirmed nephritis, and response to conventional immunosuppression therefore distinguishes this case from those previously described.

## 4 Conclusion

This case emphasizes the importance of considering SLE in a young patient presenting with atypical features such as unilateral loculated pleural effusion, once common differentials have been excluded. Early onset hypertension, especially in females, should prompt evaluation for secondary causes, including lupus nephritis. This unusual presentation of unilateral loculated pleural effusion and undiagnosed lupus nephritis underscores the subtle presentation of SLE. Early serologic evaluation in such atypical presentations may help prevent diagnostic delay, ensuring optimal treatment and contributing to the expanding clinical spectrum of this disease.

## Author Contributions

**Amrit Tripathi:** conceptualization, writing – original draft, project administration. **Ujwal Sah:** conceptualization, writing – original draft, project administration. **Saurav Shah:** methodology, data curation. **Shivaditya Singh:** methodology, data curation. **Surya Raj Nishad:** writing – review and editing.

## Funding

The authors have nothing to report.

## Disclosure

De‐identification: All clinical details, radiological images, and histopathological images have been de‐identified. No patient names, hospital identification numbers, dates of admission, or other directly identifying information appear in the text, tables, or figures, and all identifying metadata have been removed from the image files.

## Ethics Statement

The Institutional Review Board of the Manipal College of Medical Sciences, Nepal does not mandate the ethical approval for writing or publication of case reports/case series; consent was obtained. Informed written consent was obtained from the patient and patient party before writing this case report.

## Consent

Written informed consent was obtained from the patient and patient party for publication of this case report and accompanying images. A copy of the written consent is available for review by the Editor‐in‐Chief of this journal on request.

## Conflicts of Interest

The authors declare no conflicts of interest.

## Data Availability

The data that support the findings of this study are available from the corresponding author upon reasonable request.
